# Cognition difference between players of different involvement toward the concrete design features in music games

**DOI:** 10.1371/journal.pone.0216276

**Published:** 2019-05-07

**Authors:** Yi-Chen Chen, Shyue-Ran Li

**Affiliations:** 1 Department of Computer Simulation and Design, Shih Chien University, Kaohsiung City, Taiwan, Republic of China; 2 Department of Visual Arts, National Pintung University, Pingtung City, Pingtung County, Taiwan, Republic of China; West Pomeranian University of Technology, POLAND

## Abstract

When designing mobile games, how to understand preferences and cognition of players is a topic worth exploring. The main objectives of this paper are to obtain design features of music games on mobile devices, and explore players’ perceptions toward music games. The results can serve as an orientation during decision-making in game design. Based on Miryoku Engineering and the Evaluation Grid Method, this study interviewed 22 frequent users to get concrete features of game design; Moreover, 210 subjects were divided into high, medium, and low involvement groups according to CIP measures, and then this study used Multiple Regression analysis to determine whether players with different levels of involvement had different perceptions of the design features of music games. The results found 44 concrete features and six original evaluations items of game design, and also discovered that there were perception differences in different involvement groups, and only two concrete design features significantly influenced all three groups: ‘Extra games to earn more points after completing levels’ and ‘Playable without internet’.

## Introduction

Mobile games are very diverse, and each game has its own characteristics. The games that players become extremely passionate about have some key attractive features. These games induce different emotions and sensory stimulations in order to keep players interested in playing the game. This study collected related papers evaluating games in the literatures, described below.

Lu and Wang [1] explored the role that addiction to online games plays in the relationship between online satisfaction and loyalty.

Mohamed and Jaafar [2] used a comprehensive evaluation method to evaluate elements of fun and education in games. Chu[3] evaluated player experience of games through a playability matrix, which was constructed from an analysis of the literature. In the study of Chen, Shen [4], “ease and convenience”, the design features of social network games in usability were found through the methods of Kansei Engineering and the concrete features included “having fun instantly” and “playing anywhere via the internet”. Grodal [5] researched how games evoked players’ much stronger emotions. Yee [6] also studied the emotional investment of players and argued that the large amount of time spent by MMORPG players implies that they invest a significant level of emotion in playing this game. Lin, Hung [7] utilized focus group interview and statistical analysis of network survey to find that driving highly involved MMORPG gamers is coming from six main reasons: satisfaction, adventure, victory, socialization ability, self-actualization, and advancement of wealth and status. Cota et al.[8] applied a questionnaire and interviews to find that motivated the elderly to play mobile games was to help in the treatment of cognitive disorders due to aging.

Most papers in the literature have focused on researching players’ emotions or perceptions [1, 2, 9, 10] and have scarcely explored player behaviors by focusing on games’ concrete design features.

Hence, this study specially selects music games on mobile devices as a case for evaluating games, trying to extract design features of music games through a series of research processes. In general, games in this genre typically challenge players’ sense of rhythm. To achieve high scores, a player has to press buttons at precise times in a sequence shown on the screen. Compared with other types of games, music games contain no violence. They are entertaining, educational[11], and easy to operate, and therefore attract a wide range of players, so they are worth studying in detail.

This study proposes a hypothesis that different design features can attract player population with different level of involvement, and designers must precisely grasp these features when developing mobile games. The objective of this paper is to explore the following two parts: 1. The design features of music games on mobile devices; 2. The cognition and preference of the different player groups toward these design features.

## Methods

In order to clarify the relationship between players’ abstract emotions and concrete design features, and to illustrate the cognition difference between different involvement players toward games, this study mainly utilized three methods to collect and analyze data.

**Fig 1:**
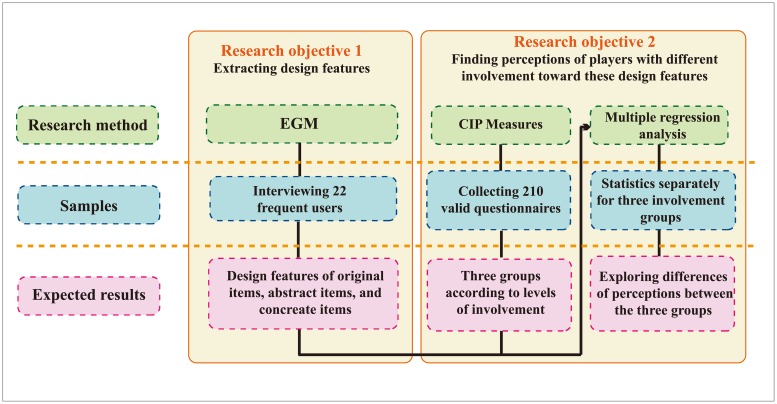
Three methods in the research process.

Fig 1 shows the research process, which indicates EGM and CIP measures were performed prior to Multiple Regression Analysis with different samplings in order to achieve two research objectives.

This study has been approved by Research Ethics Committee of National Taiwan University. The approval number is 201706ES011. The written forms of consent for interviews and questionnaire are obtained. The details of all methods are explained as follows.

### 1. Evaluation Grid Method

This study utilized EGM to extract concrete design features of music games. EGM is to collect and organize data with the methods of in-depth interviews and drawing of diagrams. It is one of research methods to transform players’ emotions into concrete design features of games.

#### (1) About Evaluation Grid Method (EGM)

Evaluation Grid Method, derived from the field of psychology, is an important research method in Miryoku Engineering to capture personal cognitive concepts and organize them. This method explicitly explores similarities or differences of objects through personal interviews with comparisons of objects, and then analyzes the characteristics of the targets [12–14]. Sanui[15] further developed this method. First, in the assessment of a target, the interviewee needs to answer the reasons of his likeness to this object.

In the related studies of Miryoku Engineering, EGM provides an approach based on theories to analyze design features of products. In order to get consumers’ feelings to the attractiveness of products, in-depth interviews are conducted with stimuli provided in the topics and comparisons of likeness which make obvious difference in interviewees’ feelings. In this way, the researcher will extract the original concepts of the topics from the interviewees and then guide the interviewees to analyze their concepts more clearly so that the concepts can be transformed into abstract emotions and concrete features for organizing real thoughts of the interviewees [16].

In other words, the process is conducted hierarchically. The manipulation steps are explained as follows:

1. First, an interviewee is given stimuli, such as oral descriptions and comparisons through pictures. The interviewee is asked to evaluate what is good or bad and what he/she likes or dislikes about the products.
2. The interviewee is asked to describe the reasons for the preference in his/her own words, and these reasons are recorded as “original evaluation items” or “middle evaluation items.”
3. The evaluation responses are recorded and the interviewee is asked for the more abstract meanings and emotions behind a response such as “upper evaluation items.”
4. We refer to the evaluation items and continue asking the interviewee for more concrete formative conditions and features of the reasons for labeling items as “lower evaluation items”.
5. All evaluation items are compiled into a diagram. The upper items are on the left, the middle ones in the middle and the lower ones on the right. All items are connected with straight lines to indicate the hierarchical relationships.

In the literature, researchers have utilised the EGM to investigate individual’s emotions and cognitions for objects and extract the exact inner decision-making factors influencing each individual in several fields, such as the design[16–22], interactive media[4, 23–26], advertisement and marketing[16, 27–29], education and psychology[30], and tourism[31, 32] fields.

In addition to exploring the emotions mobile games can invoke in players, which has been performed in previous researches, this study aims to transform these emotions into concrete design features with a systematical method, or, in other words, to discover successful features of games. The results can be used as a reference for game designers in the future.

#### (2) The operating process

This study recruited 10 researchers who had playing experience in music games and had received research interview training; 6 were male, and the researchers were aged between 23 and 41 years. They were employed to find frequent users of music games in Taiwan to interview. The requirement to be frequent users for interviews is playing any music game every week for more than half year.

First, the interviewees were asked to recall and talk about their experiences playing music games. They then opened the game app using a mobile device for reviewing the game design features. The actual operation can avoid flaws of biasing caused from that interviewees merely relied memory to answer questions[33]. Then, the interviewees were asked to talk about the elements attracted them.

For example, the interviewers asked, ‘Compared with other games, which design features in this game attract you?’ After the interviewees had responded, the interviewers continued to ask about specific reasons and the hidden, abstract items regarding the interviewees’ feelings.

An experienced interviewer would induce the interviewee to express more deep-seated preferences and feelings under the objective conditions of not deliberately guiding the interviewee[34].

Because data were collected using in-depth interviews, which are time consuming, a large-scale survey was not feasible[17, 29]. This study interviewed 22 frequent users of music games; 11 were male, 11 were female, and they were aged between 20 and 45 years.

**Fig 2:**
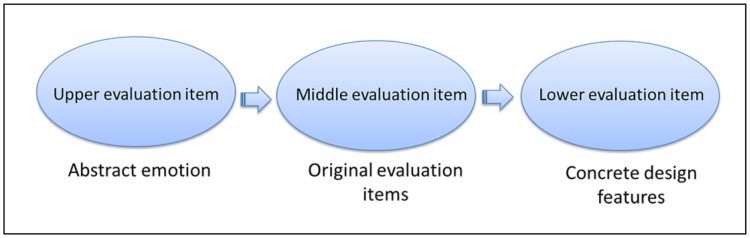
Formation of EGM.

Next, in-depth interview results related to the music games were compiled into a diagram. The upper items are on the left, the middle items in the middle, and the lower items on the right. Items connected with straight lines indicate hierarchical relationships. This arrangement constituted the EGM diagram, which integrates the data, and determines the relationships among the upper items (abstract emotions), middle evaluation items (original evaluation items), and lower evaluation items (concrete design features), which is presented in Fig 2.

### 2. CIP measures

This study adopted the consumer involvement profile proposed by Kapferer and Laurent[35] as a basic form to measure involvement and designed questions coordinating the characteristics of mobile games to determine groups of different involvement. Finally, we considered that the concrete design features may have different significance and meaning to players with different levels of involvement in different game types.

Consumer involvement profiles measure the level of relevance between games and players through questions, and a 5- point Likert scale was adopted to assess the following 13 questions regarding level of involvement:

1. Playing mobile games is important in my life.
2. Playing mobile games makes me feel happy and contented.
3. I can be very anxious if I cannot find a phone or tablet to play a game.
4. I am very anxious if I cannot find a mobile game I like.
5. I often pay to purchase items or points in mobile games.
6. I spend more than an hour per day playing mobile games.
7. I pay attention to news related to mobile games.
8. I know all the functions and characteristics of the mobile games I play.
9. Playing mobile games gives me a sense of achievement.
10. Getting a high score, rankings, and items in a game are important to me.
11. I love discussing the topic of playing mobile games with my friends.
12. I care what people think about the fact that I play mobile games.
13. I can find out other people’s preferences from the mobile games they play.

This study grouped involvement levels using the quartile method based on the players’ total scores in assessment questions of involvement. The players were divided into three groups: high involvement (first 25%), medium involvement (middle 50%), and low involvement (last 25%). The total score was obtained from the degree of the subjects’ agreement in every assessment question (1 to 5). Every subject’s 13 scores were summed.

### 3. Multiple regression analysis

In the first method, aforementioned EGM obtained original evaluation items and concrete design features. We converted these into a questionnaire with measurable questions and that could be distributed to a large number of game players. We used a 5-point Likert scale to assess the subjects’ degree of agreement.

For examples: “The games have favourable level design because of providing a teaching mode”; “The games have favourable level design because of ability to choose difficulty or speed of levels”. The subjects gave 1~5 points for all original evaluation items and concrete design features according to their agreement.

Besides, in the same questionnaire, this study used the second method, CIP measures, to identify three involvement groups which each subject was belong to.

Finally, this study used the third method, statistics of multiple regression analysis[36] via SPSS, to explore how the variables of the three involvement groups are affected by the variables of concrete design features of music games.

## Results

### 1. Results of EGM

**Fig 3:**
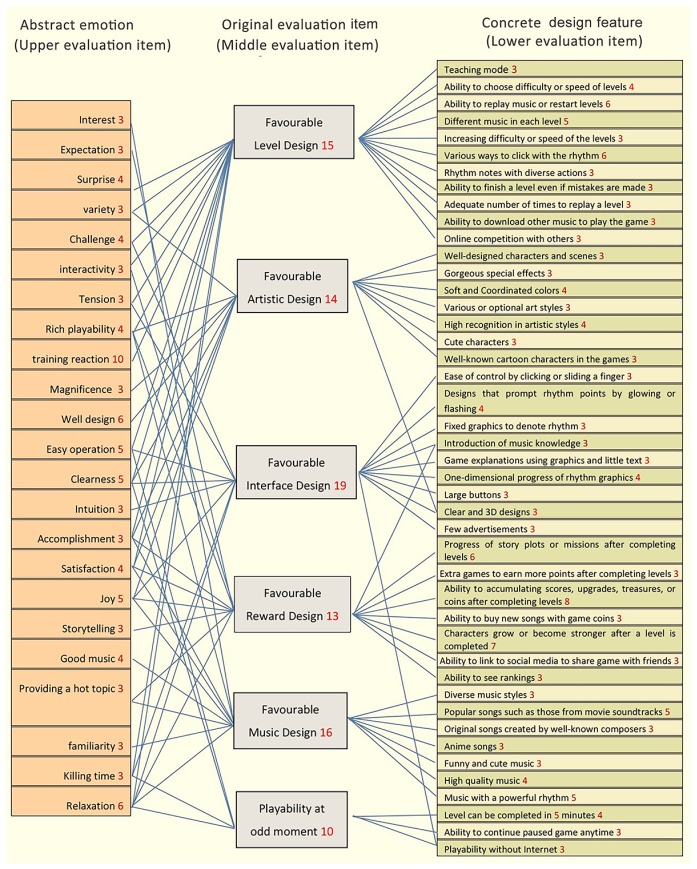
EGM of music games.

This study individually produced EGM diagrams for the 22 interviewees’ data, and then integrated the 22 diagrams into one, showed in Fig 3. The integrated EGM diagram showed 44 concrete design features and 6 original evaluations items. We can clearly see why the interviewee is attracted by a particular event after completing this step.

These items were obtained using qualitative research methods. However, this study also required quantitative research methods to verify these items. Therefore, we converted 44 concrete design features and 6 original evaluations items into a questionnaire with measurable questions; 210 valid questionnaires were collected.

### 2. Results of questionnaire

#### (1) Demographic characteristics

The construct validity of the 44 measurable questions was analysed. The Kaiser–Meyer–Olkin (KMO) measure obtained was 0.92, and the Bartlett’s test of sphericity score was 5094.96, which indicated relatively high validity. In the reliability analysis, the Cronbach’s alpha was 0.95, indicating that the questionnaire had high reliability. The demographics of the respondents are presented in Table 1.

**Table 1:** Socio-demographic information.

		Number
Gender	Male	127
	Female	83
Age	10–19	147
	20–29	50
	30–39	8
	40–49	5
Education	Elementary school	33
	Junior high school	2
	Senior high school	82
	College or University	89
	Graduate school	4
Profession	Student	190
	Government employee	15
	Service	4
	Manufacturing	1
	**Total**	**210**

#### (2) Grouping according to involvement levels

The total score was obtained from the degree of the subjects’ agreement in every assessment question (1 to 5). Every subject’s 13 scores were summed. The highest sum obtained for the questionnaire was 61 points, and the lowest sum was 13. The involvement grouping results are shown in Table 2.

**Table 2:** Grouping of involvement.

Sum of score	Involvement	Number of subjects
**13–25**	*LI* (Low involvement)	51
**26–42**	*MI* (Medium involvement)	105
**43–61**	*HI* (High involvement)	54

#### (3) Results of multiple regression analysis

**Fig 4:**
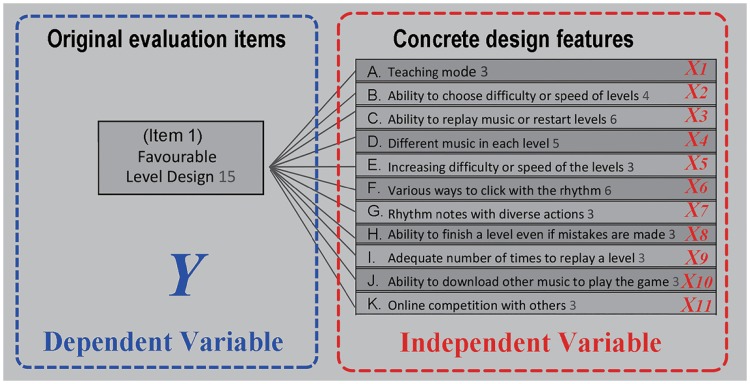
Example of the first item for concept of multiple regression analysis.

In EGM, an original evaluation item is a goal those concrete design features have to achieve. For example of the first evaluation item in Fig 4, in order to make players feel “favourable level design”, designers can strengthen the features, “the teaching mode”, “the ability to choose difficulty or speed of levels”, “the ability to replay music or restart levels”, and so on.

In the perceptions of these three involvement groups, is there any design feature which has linear relationship in these 6 original evaluation items? In another word, to improve these six items efficiently, which design feature do designers have to strengthen?

This study conducted multiple regression analysis separately with the three groups to determine how the three involvement groups were affected by 44 concrete design features in the six original evaluation items. In the multiple regression analysis, the dependent variables were subjects’ scores (1–7) of the importance of the original evaluations items in the questionnaires, and the independent variables were their degree of agreement (1–5) for each concrete design feature (see Fig 4). There were six items and three involvement groups; thus, 18 multiple regression analyses were performed in total (6 items * 3 groups). The multiple regression analysis results for the three groups are shown in Tables 3, 4 and 5, respectively.

**Table 3:** Multiple regression analysis with *LI*.

Original Evaluations Items	Concrete Design Features with Significant Impact	Constant	B Value	Beta (β)	T Value	Sig.	Mean/SD
***Item 1*. *Favourable Level Design***	E. Increasing difficulty or speed of the levels	1.945	.788	.482	3.846	.000***	3.014 1.236
***Item 2*. *Favourable Artistic Design***	C. Soft and coordinated colours	.974	1.026	.789	8.982	.000***	3.087 1.314
***Item 3*. *Favourable Interface Design***	A. Ease of control by clicking or sliding a finger	.789	.557	.415	3.240	.002**	3.168 1.253
	G. Large buttons		.492	.375	2.292	.005**	
***Item 4*. *Favourable Reward Design***	B. Extra games to earn more points after completing levels	1.227	.881	.138	6.387	.000***	3.181 1.278
***Item 5*. *Favourable Music Design***	C. Original songs created by well-known composers	1.878	.841	.643	5.881	.000***	3.302 1.278
***Item 6*. *Playable at odd moments***	C. Designs playable without Internet	2.345	.772	.611	5.402	.000***	3.480 1.324

*P < 0.0

** P <0.0

*** P < 0.001

**Table 4:** Multiple regression analysis with *MI*.

Original Evaluations Items	Concrete Design Features with Significant Impact	Constant	B Value	Beta (β)	T Value	Sig.	Mean/SD
***Item 1*. *Favourable Level Design***	E. Increasing difficulty or speed of the levels	3.108	.432	.328	3.511	.001**	3.668 1.028
	J. Ability to download other music to play game		.189	.187	1.998	0.048*	
***Item 2*. *Favourable Artistic Design***	E. High recognition in artistic styles	2.513	.418	.304	3.314	.001**	3.656 1.042
	F. Cute characters		.323	.292	3.184	.002**	
***Item 3*. *Favourable Interface Design***	F. One-dimensional progress of rhythm graphics	2.899	.321	.254	2.491	.014*	3.655 0.949
	B. Designs that prompt rhythm points using glowing or flashing		.316	.239	2.343	.021*	
***Item 4*. *Favourable Reward Design***	E. Characters grow or become stronger after a level is completed	1.963	.533	.399	4.574	.000***	3.724 0.990
	B. Extra games to earn more points after completing levels		.330	.233	2.668	.009**	
***Item 5*. *Favourable Music Design***	A. Diverse music styles	.746	.691	.481	5.637	.000***	3.823 1.049
	G. Music with powerful rhythms		.309	.233	2.614	.010*	
	E. Funny and cute music		.229	.186	2.253	.013*	
***Item 6*. *Playable at odd moments***	B. Ability to continue paused game anytime	1.654	.507	.320	3.356	.001***	3.998 1.016
	C. Designs playable without Internet		.421	.304	3.195	.002**	

* P < 0.0

** P <0.01

*** P < 0.001

**Table 5:** Multiple regression analysis with *HI*.

Original Evaluations Items	Concrete Design Features with Significant Impact	Constant	B Value	Beta (β)	T Value	Sig.	Mean/SD
***Item 1*. *Favourable Level Design***	G. Rhythm notes with diverse actions	2.481	.455	.273	2.105	.04*	4.029 0.993
	A. Teaching mode		.316	.261	2.015	.049*	
***Item 2*. *Favourable Artistic Design***	B. Gorgeous special effects	2.656	.674	.413	3.273	.002*	3.962 1.011
***Item 3*. *Favourable Interface Design***	A. Ease of control by clicking or sliding a finger	1.809	.886	.528	4.484	.000***	3.881 0.967
***Item 4*. *Favourable Reward Design***	B. Extra games to earn more points after completing levels	3.466	.521	.438	3.515	.001***	3.984 1.032
***Item 5*. *Favourable Music Design***	C. Original songs created by well-known composers	4.386	.432	.325	2.475	.017*	4.082 0.957
***Item 6*. *Playable at odd moments***	C. Designs playable without Internet	2.949	.609	.552	4.773	.000***	4.171 0.945

* P < 0.05

** P <0.01

*** P < 0.001

## Discussion

The schedule and manpower of this study are finite, so there are the following research limitations:

1. When we interviewed a frequent user, he/she could only provide ideas or feelings for his/her familiar games. This study has sought as many interviewees as possible, but still may not be able to cover all music games.
2. VanVoorhis and Morgan [37] recommended an absolute minimum of 10 participants per predictor variable for regression using six or more predictors. In this study, number of predictors is 44, so the ideal number of the subjects is supposed to be 440. However, the subjects of the questionnaires must be the players who had used music games, which needed to be screened strictly and the number was limited. Moreover, according to Cohen [38], we calculated the number of samples using the medium effect size f^2^ = 0.15, α = 0.05, and maintaining the power = 0.8, and determined the number of subjects needed for the questionnaire was about 210 ultimately.

This section presents explanation of the six original evaluation items in EGM and discusses the common concrete design features that different involvement groups have significance.

### 1. The results of EGM

The EGM diagram in Fig 3 displays the detailed results of the interviews.

#### (1) Favourable level design

In this item, there were 11 concrete design features in which most players expressed a preference, including ‘A. Teaching mode’, ‘B. Ability to choose difficulty or speed of levels’, ‘C. Ability to replay music or restart levels’, etc. The abstract reasons in this item were ‘surprise’, ‘variety’, ‘challenge’, etc.

These results agree with the design principles of casual games suggested by Greechan[39]. Game play complexity is carefully designed and gradually introduces players to higher levels. Greechan also mentioned that forgiving, nonpunishing gameplay provides numerous hints before a punishment is given and encourages players to continue attempts to increase their sense of accomplishment. In addition, various ways of clicking with the rhythm and designs that enable players to choose levels of difficulty or speed make games flexible and open. Music games are thus similar to casual games in their level design, and these games should take players of different abilities into account using these methods.

#### (2) Favourable artistic design

In this item, there were seven concrete design features in which most players expressed a preference, including ‘A. Well-designed characters and scenes’, ‘B. Gorgeous special effects’, ‘C. Soft and coordinated colours’, etc. The abstract reasons in this item were fine images, gorgeous special effects, and beautiful backgrounds, showing rich content to create visual excitement, portray high quality, and offer comfort.

#### (3) Favourable Interface Design

In this item, there were 10 concrete design features for which most players expressed a preference, including ‘A. Ease of control by clicking or sliding a finger’, ‘B. Designs that prompt rhythm points by glowing or flashing’, ‘C. Fixed graphics to denote rhythm’, etc. The abstract reasons in this item were ‘challenge’, ‘tension’, ‘easy operation’, etc. These indicate that the interface features of attractive music games include the following: (a) glowing or flashing rhythm notes are clear and easy to identify, (b) illustration not text for game descriptions, and (c) advertisements in games are few. These features generate relaxing visual effects for game players and facilitate game playing.

#### (4) Favourable reward design

In this item, there were eight concrete design features for which most players expressed a preference, including ‘A. Introduction of music knowledge’, ‘B. Progress of story or missions after completing levels’, ‘C. Extra games to earn more points after completing levels’, etc. The abstract reasons in this item were ‘expectation’, ‘interactivity’, ‘rich playability’, etc. These include internal and external rewards:

1. Internal rewards: Scores, gold coins, mileage, health points, and lucky draw mechanisms are the internal rewards in general games and are also effective rewards in music games. Making characters stronger and progressing the story or mission are also features of music games. In the upper items, these mechanisms prompt players to feel a sense of accomplishment at their overcoming of challenges, and storytelling factors are also crucial characteristics of some music games.
2. External rewards refer to players’ competition over rankings, and rankings are even shared with friends in the community. This result agrees with those of Campbell[40] and Radoff[41] regarding game communities attracting players to discuss and share their game experiences, achievements, and photos with friends.

#### (5) Favourable Music Design

In this item, there were seven concrete design features for which most players expressed a preference, including ‘A. Diverse music styles’, ‘B. Popular songs such as those from movie soundtracks’, ‘C. Original songs created by well-known composers’, etc. The abstract reasons in this item were ‘interest’, ‘rich playability’, ‘accomplishment’, etc. Players prefer to participate in games with diverse familiar music of different styles, which produces pleasure and a high sense of accomplishment. Listening pleasure obtained from loving and enjoying music is the main value of music games.

#### (6) Playable at odd moments

In this item, there were three concrete design features for which most players expressed a preference, including ‘A. Level can be completed in 5 minutes’, ‘B. Ability to continue paused game anytime’, and ‘C. Playability without internet’. The abstract reasons in this item were ‘easy operation’, ‘killing time’, ‘relaxation’, etc.

Music games are easy to start, pause, leave, and return to[42] because they generally do not require Internet connectivity. Thus, players can choose the time in which they play music games. Besides, the length of a song is generally only 3–5 minutes, so players can quickly know whether they pass levels or not. Therefore, it is critical to allow players short bursts of enjoyment.

### 2. The results of statistical analysis

Tables 3, 4 and 5 were combined to obtain Table 6, which compares the multiple regression results of *HI*, *MI* and *LI*. Table 6 shows the concrete design features having significant levels of impact on different involvement groups.

**Table 6:** Comparison of the concrete design features reaching significant levels to impact different involvement groups.

Original Evaluation Items	*HI*	*MI*	*LI*
***Item 1*. *Favourable Level Design***	G. Rhythm notes with diverse actions		
	A. Teaching mode		
		E. Increasing difficulty or speed of the levels	E. Increasing difficulty or speed of the levels
		J. Ability to download other music to play game	
***Item 2*. *Favourable Artistic Design***	B. Gorgeous special effects		
		F. Cute characters	
		E. High recognition in artistic styles	
			C. Soft and Coordinated colours
***Item 3*. *Favourable Interface Design***	A. Ease of control by clicking or sliding a finger		A. Ease of control by clicking or sliding a finger
		F. One-dimensional progress of rhythm graphics	
		B. Designs that prompt rhythm points using glowing or flashing	
			G. Large buttons
***Item 4*. *Favourable Reward Design***	B. Extra games to earn more points after completing levels	B. Extra games to earn more points after completing levels	B. Extra games to earn more points after completing levels
		E. Characters grow or become stronger after a level is completed	
***Item 5*. *Favourable Music Design***	C. Original songs created by well-known composers		C. Original songs created by well-known composers
		A. Diverse music styles	
		G. Music with powerful rhythms	
		E. Funny and cute music	
***Item 6*. *Playable at odd moments***	C. Playable without Internet	C. Playable without Internet	C. Playable without Internet
		B. Ability to continue paused game anytime	

Two features reached the level of significance among all three groups: ‘B. Extra games to earn more points after completing levels’ in Item 4, and ‘C. Playable without Internet’ in Item 6.

The ability to play a game offline is crucial for players. Music games are easy to start, pause, leave, and return to[42] because they generally do not require Internet connectivity. Thus, players can choose the time in which they play music games, with them able to freely leave and return to the game. These two concrete design features, ‘ability to continue paused games anytime’ and ‘playable without Internet’ are concrete methods of enabling players relax when playing music games.

Some music games provide short extra games after the completion of levels, which allow players to earn more coins or points, giving players a sense of accomplishment and challenge. One feature significantly influenced the common cognition in the *MI* and *LI*: ‘E. Increasing difficulty or speed of the levels’ in Item 1.

Moreover, two features significantly influenced the common features in the *HI* and *LI*: ‘A. Ease of control by clicking or sliding a finger’ in Item 3, and ‘C. Original songs created by well-known composers’ in Item 5.

## Conclusion

This study discovered that only two concrete design features significantly influenced all three groups: ‘Extra games to earn more points after completing levels’ and ‘Playable without Internet’.

Relevant literature on the involvement and online games has revealed that player groups at different levels of involvement have different perceptions towards the same product. The experiment conducted in this study yielded similar results. Groups with different levels of involvement in music games had different preferences of game design features.

This study not only investigated players’ abstract emotions and perceptions, but also transformed those into concrete design features in game designs. These design features offer a concrete method for improving music games. Designers can strengthen specific design features according to their different targets in the gaming market.

## Supporting information

S1 File Original data for statistics. (XLSX)
